# The impact of remote consultations on brief conversations in general practice

**DOI:** 10.3399/BJGPO.2021.0199

**Published:** 2022-03-23

**Authors:** Faraz Mughal, Helen Atherton, Hassan Awan, Tom Kingstone, Aaron Poppleton, Victoria Silverwood, Carolyn A Chew-Graham

**Affiliations:** 1 Unit of Academic Primary Care, Warwick Medical School, University of Warwick, Coventry, UK; 2 School of Medicine, Keele University, Keele, UK

**Keywords:** Remote consultations, Brief conversations, International, General practice, Primary healthcare

## Background

The consultation between the family doctor and patient is the cornerstone of general practice, supporting relationship-based care and continuity: which can improve patient outcomes.^1,2^ It enables rapport to be established and provides a setting to begin conversations on sensitive topics such as weight management in a patient with osteoarthritis, or alcohol consumption in someone with elevated liver function tests. Brief conversations in general practice are introductory enquiries or exchanges that are initiated by the GP, often as a stepping stone to more detailed discussions, to improve patient’s capability to make positive changes for their health and wellbeing.

**Clinical encounter with family**

FM recollects a face-to-face consultation in 2016 as a GP, where after handling the initial presenting problem of the child, he spoke to the parents about the child’s diet. He discovered that the parents provided the child with fast food (chicken and chips) each day for dinner after school. The parents were not aware that this was detrimental to their child’s health. FM used this disclosure to provide brief advice on the importance of a balanced diet and offered some low-cost, easy-to-prepare alternatives.

Dealing with and managing the patient’s presenting complaint is important, but using the opportunity to explore aspects of health promotion, prevention, and behaviour change is key to realising the exceptional potential of the primary care consultation.^3^ Leaning on the *Making Every Contact Count* public health initiative, GPs can use consultations to initiate brief conversations, which in turn can lead to the delivery of interventions and potentially result in long-lasting benefits for patients and the NHS.^4,5^

In a consultation with someone who is experiencing distress GPs who utilise an open questioning style and sensitively ask about self-harm can facilitate further discussion about self-harm and suicide: this can be life-saving.^6^ In the management of obesity, language is important and seeking the permission of the patient — *‘Would you mind if we spoke about your weight?’*, for example — provides a chance for patients to share concerns or ask questions.^7^ Patients perceive receiving a brief health behaviour change intervention as appropriate and helpful, especially in long term conditions, with the doctor-patient relationship the foundation of the conversation.^8^

In this commentary we discuss how the increased reliance on remote consulting, exacerbated by COVID-19, may impact on the opportunity for brief conversations in general practice globally, and outline implications for clinical practice and policy, and suggestions for future research.

### Remote consulting and COVID-19

The delivery of general practice consultations across countries has changed substantially because of COVID-19.^9,10^ Many countries increased their use of remote consultations (telephone, video, email, and other forms of internet communication) to continue to provide essential primary care services while mitigating the risk of COVID-19 in staff and patients.^10^ Across one clinical commissioning group in England, there was a reduction in GP consultations (*n* = 180) per 1000 patients early in the COVID-19 pandemic (April 2020), 89% of which were telephone consults, compared to 218 GP consultations per 1000 reigstered patients, where 31% of consultations were by telephone, in 2019.^11^ There were no video consultations recorded in 2019, whereas in 2020 1% (all age groups) were coded as video consultations, rising to 3% for patients aged >85 years in 2020 and fewer than 1% of were recorded as e-consultations.^11^ For many primary care clinicians, this shift required on-the-job learning and engagement with new technologies as they came to rely on these alternative approaches in a short space of time.^12^

## Communication in remote consultations

Different communication mediums have different strengths and weaknesses as modes of consultation. Telephone consultations are acceptable to both GPs and patients, and are seen as an appropriate consultation mode for non-complex, single concerns such as new acute problems, where patients may be asked to attend later for a face-to-face consult.^13^ The subtle features observable during a face-to-face consultation, such as body language (sitting uncomfortably or looking away) and non-verbal cues (a wince or a facial reaction), are lost in a telephone consultation, however.^14^ Patients who perceive that they don’t communicate well described a lack of trust in telephone consultations and preferred to see a doctor face-to-face, where they may be able to better articulate their problems.^15^

Video consultations result in varied experiences for patients, reflecting the complexity of using these in practice. Research has shown that patients can encounter difficulty in establishing a doctor–patient relationship using video, feel rushed, and have trouble with turn taking.^16^ Conversely, when used as a method of follow up in the context of an existing doctor–patient relationship, patients appreciate the visual element of a video consultation over and above what is achievable in a telephone consultation.^17^

A recent systematic review found that primary care clinicians described concerns with e-consultations (email or online messaging) around the potential negative effect on the doctor–patient relationship, due to the need for GPs to use non-technical language; however, the ability to communicate immediately and in some patients, their access to notes, enhanced trust and communication, and strengthened the therapeutic relationship.^18^

These findings demonstrate it is crucial to develop doctor–patient rapport when using a new medium and this in turn can enable brief conversations to occur. If FM undertook a telephone or e-consultation in the above consultation, he would not have seen the child’s size or perhaps the parents would not have felt comfortable talking about food habits, and therefore the opportunity for the brief conversation may have been lost.

### Missed conversations are missed opportunities

To maximise the opportunity for brief conversations, establishing GP–patient rapport in remote consultations is crucial. Missed brief conversations are missed chances for promoting good health and well-being in general practice. In the long term, losing the opportunity for brief conversations may impact on patient populations, especially in those with long-term conditions where the potential for positive change may be greatest, and subsequently negatively burden primary care services and systems. This requires ongoing evaluation as remote consulting becomes more established in general practice.

## Implications for practice, policy, and future research

The capability of clinicians to adopt and confidently use remote consultation methods, which most GPs have not trained in, cannot be assumed and evidence-informed training will need upscaling. In addition, GPs need to be aware that consulting remotely may affect rapport-building and should consider this when initiating brief conversations. Policymakers need to contemplate the growing evidence on relationship-based care and ensure this is protected and encouraged in a COVID-19 context with growing time constraints: this should be prioritised by primary care systems worldwide.

Retaining relationship-based care at the heart of the general practice consultation will enable brief conversations to continue for the benefit of patients, the public, clinicians, and health services. Future research should prioritise how the range of remote consultation options can support patients in their ongoing care. Conversation analyses across different modes of remote consultations would enable an in-depth examination of how brief conversations occur. The views of patients and clinicians on remote brief conversations need to be explored to establish what elements would be acceptable and useful, therefore informing best practice recommendations for general practice.

## Notes

HA spoke on relationship-based care at RCGP Annual Conference 2021, and is a member of the Evaluation Steering Committee, Digital First Primary Care, NHS England.
